# Comparing the diagnostic performance of radiotracers in prostate cancer biochemical recurrence: a systematic review and meta-analysis

**DOI:** 10.1007/s00330-022-08802-7

**Published:** 2022-04-29

**Authors:** Weili Ma, Jiwei Mao, Jianfeng Yang, Ting Wang, Zhen Hua Zhao

**Affiliations:** 1 Department of Radiology, Shaoxing People’s Hospital (Shaoxing Hospital, Zhejiang University School of Medicine), Key Laboratory of Functional Molecular Imaging of Tumor and Interventional Diagnosis and Treatment of Shaoxing City, Shaoxing, 312000 China; 2 Department of Radiotherapy, Shaoxing People’s Hospital (Shaoxing Hospital, Zhejiang University School of Medicine), Shaoxing, 312000 China

**Keywords:** Recurrence, Prostatic neoplasms, Positron emission tomography, Meta-analysis

## Abstract

**Objectives:**

To systematically assess the early detection rate of biochemical prostate cancer recurrence using choline, fluciclovine, and PSMA.

**Methods:**

Under the guidance of the Preferred Reporting Items for Systematic reviews and Meta-Analysis Diagnostic Test Accuracy guidelines, literature that assessed the detection rates (DRs) of choline, fluciclovine, and PSMA in prostate cancer biochemical recurrence was searched in PubMed and EMBASE databases for our systematic review from 2012 to July 15, 2021. In addition, the PSA-stratified performance of detection positivity was obtained to assess the DRs for various methods, including fluciclovine, PSMA, or choline PET/CT, with respect to biochemical recurrence based on different PSA levels.

**Results:**

In total, 64 studies involving 11,173 patients met the inclusion criteria. Of the studies, 12, 7, and 48 focused on choline, fluciclovine, and PSMA, respectively. The pooled DRs were 24%, 37%, and 44%, respectively, for a PSA level less than 0.5 ng/mL (*p* < 0.001); 36%, 44%, and 60% for a PSA level of 0.5–0.99 ng/mL (*p* < 0.001); and 50%, 61%, and 80% for a PSA level of 1.0–1.99 ng/mL (*p* < 0.001). The DR with ^18^F-labeled PSMA was higher than that with ^68^Ga-labeled PSMA, and the DR was 58%, 72%, and 88% for PSA levels < 0.5 ng/mL, 0.5–0.9 ng/mL, and 1.0–1.99 ng/mL, respectively.

**Conclusion:**

The DRs of PSMA-radiotracers were greater than those of choline-radiotracers and fluciclovine-radiotracers at the patient level. ^18^F-labeled PSMA achieved a higher DR than ^68^Ga-labeled PSMA.

**Key Points:**

*• The DRs of PSMA-radiotracers were greater than those of choline-radiotracers and fluciclovine-radiotracers at the patient level.*

*•* ^*18*^*F-labeled PSMA achieved a higher DR than* ^*68*^*Ga-labeled PSMA.*

## Introduction

Prostate cancer (PCa) is the most common malignancy of the male genitourinary system worldwide [1]. Radical prostatectomy or radiation therapy remains the most widely used treatment for localized PCa with intermediate and high risks [2]. After definitive therapy, biochemical recurrence (BCR) of PCa still recurs approximately 39–41% of the time [3, 4]. At this stage of the disease, it is still essential to define the location and extent of metastasis and initial recurrence to help urologists make further treatment plans [5]. The detection of subtle or occult recurrence and metastasis after treatment continues to pose a challenge [6]. In this setting, PET/CT is particularly superior to conventional imaging modalities such as CT (computed tomography)/MRI (magnetic resonance imaging) because of its higher detection rate (DR) for low-volume metastatic or locally initial recurrence [7, 8].

To date, of the radiolabels that have been evaluated, 11C-choline and 18F-fluciclovine were approved by the Food and Drug Administration (FDA) in 2012 and 2016 [9]. Prostate-specific membrane antigen (PSMA), a cell surface protein, is highly expressed in the majority of PCa cells and in PCa recurrence [10, 11]. ^68^Ga- and ^18^F-labeled PSMA are promising new radiotracers for detecting the BCR of PCa and radio-nuclear therapy. [^68^Ga]Ga-PSMA-11 was the first PSMA PET tracer that was approved by the FDA [6]. Moreover, ^18^F-labeled PSMA agents have also been employed in clinical practice.

Previous evidence-based studies have compared the diagnostic performance of choline, fluciclovine, and PSMA PET/CT in PCa patients with BCR, and particularly at a PSA level less than 2 ng/mL^6^. However, they only concentrated on long-half radionuclides as ^18^F-labeled PET tracers and compared their diagnostic performance in detecting patients with BCR. To our knowledge, a comprehensive comparative meta-analysis of choline, fluciclovine, and PSMA for detecting PCa patients with BCR and low PSA levels has not been performed. Furthermore, several studies have shown that the higher image resolution of ^18^F, as a longer half-life nuclide, is slightly better than that of ^68^Ga [12, 13]. However, evidence-based data based on ^18^F-labeled and ^68^Ga-labeled PSMA are still lacking. Therefore, the aims of this meta-analysis were to compare the DRs of radiotracers, including choline, fluciclovine, and PSMA PET/CT, for biochemical recurrence with PSA levels less than 2 ng/mL and to perform subgroup analyses based on ^18^F-labeled and ^68^Ga-labeled PSMA.

## Materials and methods

### Search strategy

This meta-analysis was performed in accordance with the PRISMA-DTA statement [14]. Two reviewers searched the library databases of PubMed and Embase that involved the DR of PET/CT using choline, fluciclovine, and PSMA agents between 2012 and July 2021. The search terms included the following: prostatic neoplasms, prostate cancer, recurrence, biochemical recurrence, ^18^F-choline, ^11^C-choline, fluoromethylcholine, [^18^F]fluciclovine, [^18^F]FACBC, [^18^F]PSMA-1007, [^18^F]DCFPyl, [^18^F]DCFBC, [^68^Ga]Ga-PSMA-11, [^18^F]PSMA-11, [^68^Ga]Ga-PSMA-I&T, [^68^Ga]Ga-THP-PSMA, [^64^Cu]Cu-PSMA-617, [^18^F]JK-PSMA-7, and [^68^Ga]Ga-HBED-CC. To expand our search, the lists of references from the retrieved articles were also checked. Two reviewers independently reviewed the references in the included studies.

### Study selection

Both retrospective and prospective studies involving males with PCa with BCR who underwent PET/CT using choline, fluciclovine, and PSMA agents between 2012 and July 2021 were included. In addition, single-arm trials, comparative, single-center, multicenter, and clinical trials were also included. Studies were excluded as follows: abstracts, comments, letters, conference records, case reports, reviews, and meta-analyses, non-English articles, studies for staging purposes, and studies assessing specific types of metastatic disease, such as that of bones or lymph nodes. If the studies included patients from the same group, the largest sample was reviewed.

### Data extraction and quality assessment

Two reviewers independently extracted and confirmed the data. Information was recorded from each study, including year of publication, radiotracer, imaging protocols, country of origin, study design (prospective or retrospective, multicenter or single center), patient age, sample size, treatment, PSA stratified into tiers (PSA level less than 0.5 ng/mL ng/mL, 0.5–0.99 ng/mL, and 1–1.99 ng/mL), and DR. We used the revised Quality Assessment of Studies of Diagnostic Accuracy, which was included in the QUADAS-2 tool for quality assessment [15]. Each item was judged as “yes,” “no,” or “unclear.” Any disagreements were resolved by consensus.

### Statistical analysis

The pooled estimates with 95% CIs were the DRs of PSA-stratified patients with biochemical recurrence after treatment. The pooled estimates for the DRs of different radiotracers were compared using a chi-square test. Forest plots with 95% confidence intervals were used to visually assess the results. The inconsistency index (*I*^2^) was used to assess statistical heterogeneity of the included studies. The Cochrane *Q* with *p* < 0.05, and *I*^2^ > 50% indicated significant heterogeneity. A random-effects model was applied, and marked heterogeneity was observed. Statistical significance was set at a *p*-value less than 0.05. Egger’s test and funnel plot tests were conducted to assess the publication bias. The open-source statistical software R was used to conduct all statistical analyses (version 3.6.3; www.r-project.org/). The QUADAS quality evaluation was conducted using RevMan (version 5.3).

## Results

### Literature search

**Fig. 1:**
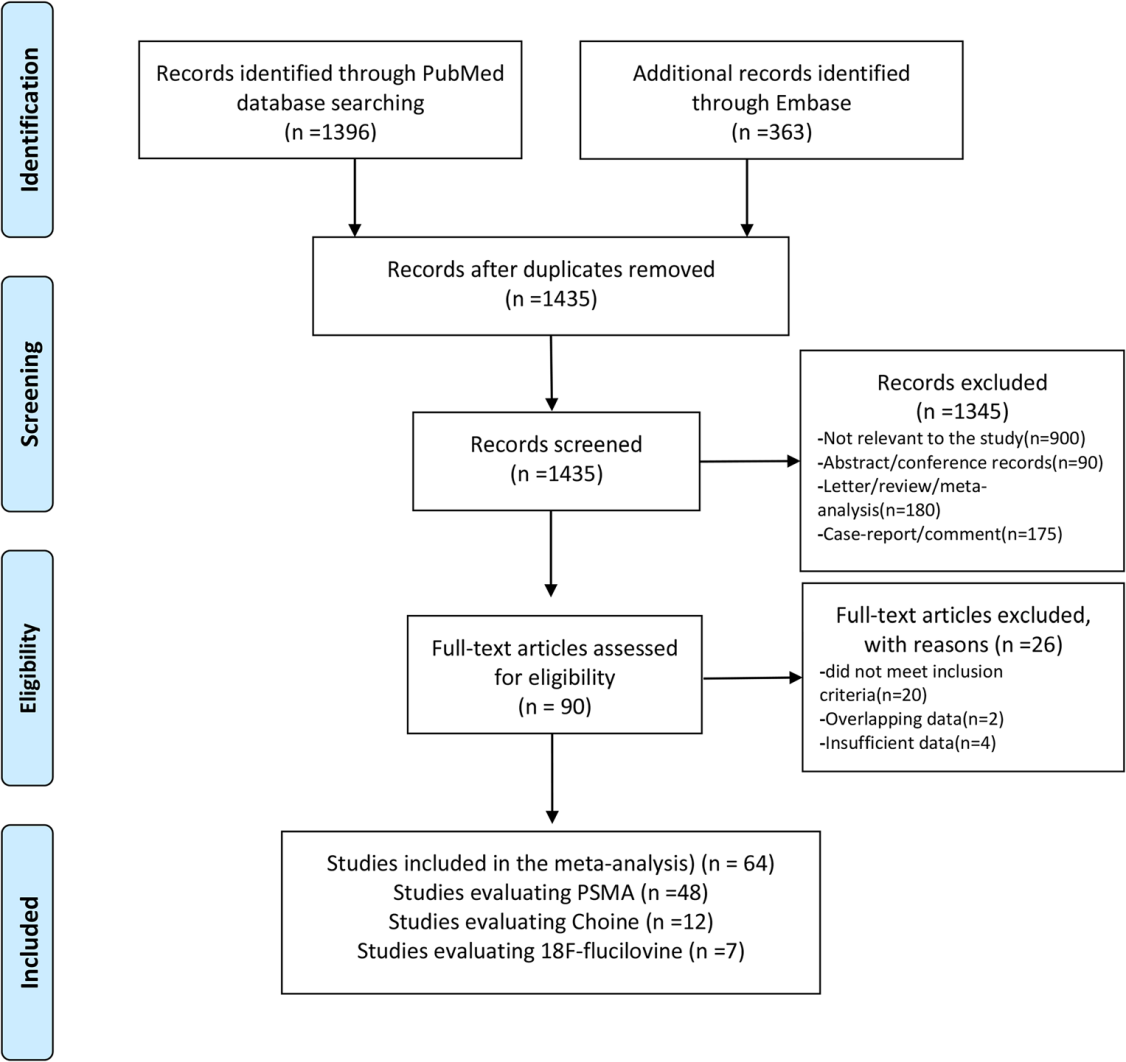
Study flow diagram

Figure 1 presents an overview of the inclusion process. Initially, 1759 total articles were identified through PubMed and Embase databases using the search terms and keywords (1396 in PubMed, and 363 in Embase). In total, 324 duplicate records were removed. After screening titles and abstracts, 1345 records were excluded; 900 because they were not relevant to the study; 90 as they were abstracts or conference records; 180 as they were letters, reviews, or meta-analyses; and 175 as they were case reports and comments.

**Fig. 2:**
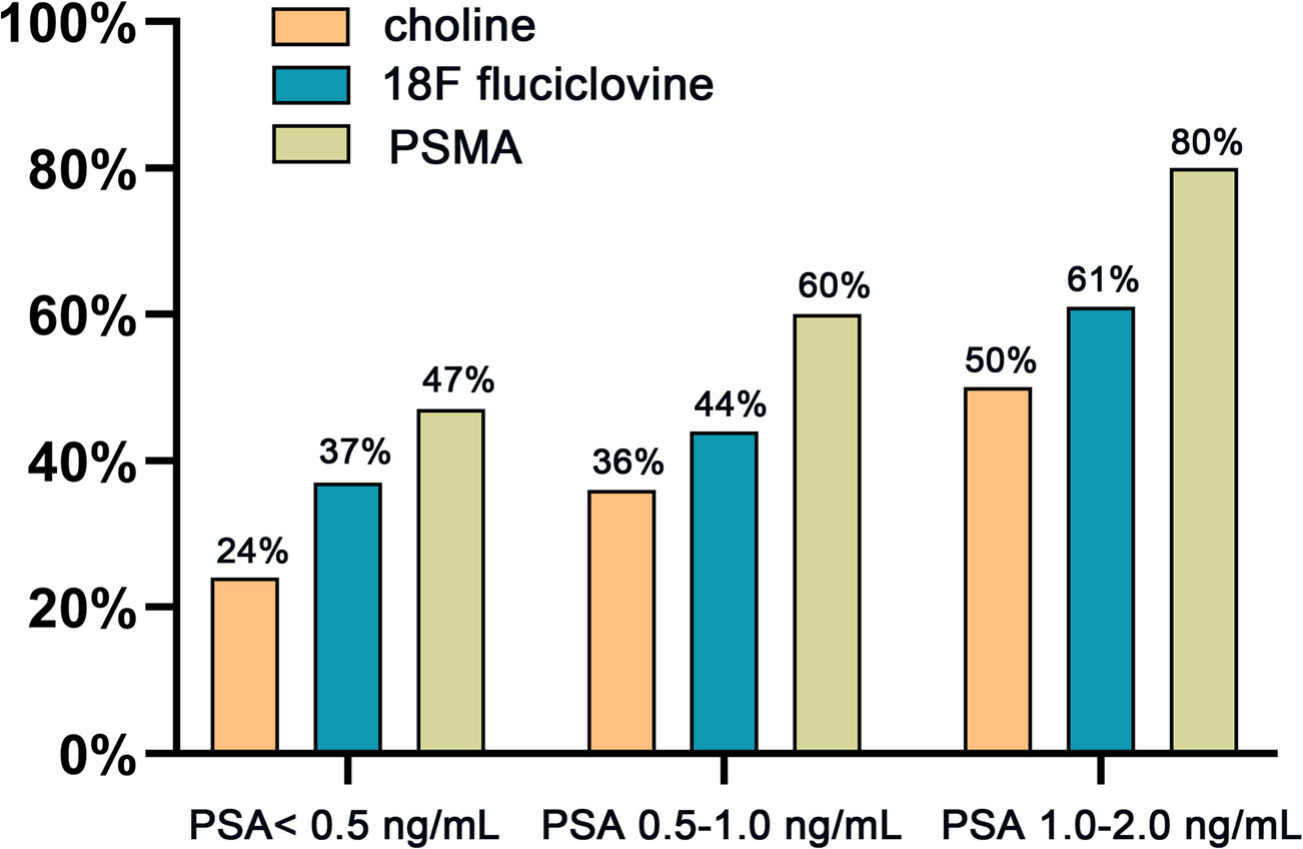
The proportion of three tracers in different PSA stratification

Of the remaining studies, 90 full-texts were reviewed, and 64 studies were included in this meta-analysis. Of these, 48 studies focused on the performance of PSMA PET/CT for patients with BCR. Seven and 12 studies were included for fluciclovine and choline PET/CT, respectively. Of these, two studies evaluated both PSMA and choline [16, 17], and one study evaluated both chorine and fluciclovine [18]. The characteristics of the included articles are presented in Tables 1, 2 and 3. Figure 2 shows the proportion of different tracers in the included studies under PSA stratification.

**Table 1:** Summary of included studies using 18F-fluciclovine

Reference	Year	Country	Radiotracer	Study design	Study population	Mean/median age (y)	No. of patients	Scanner
Nanni [18]	2015	Italy	[^18^F]fluciclovine	Prospective/SC	RP	69 (55–83)	89	Discovery STE (GE)
Odewole OA [19]	2016	America	[^18^F]fluciclovine	Retrospective/SC	Mixed	67 ± 8	53	Discovery DLS 690 PET/CT (GE)
Akin-Akintayo [20]	2017	America	[^18^F]fluciclovine	Prospective/SC	RP	62 ± 7.54	42	Discovery MV690 PET/CT (GE)
Bach-Gansmo T [21]	2017	Norway	[^18^F]fluciclovine	Retrospective/MC	RP	67 (42–90)	596	Various (site dependent)
England [22]	2019	America	[^18^F]fluciclovine	Retrospective/SC	Mixed	67 (53–77)	28	PET/CT (Siemens)
Andriole [23]	2019	America	[^18^F]fluciclovine	Prospective/MC	Mixed	67 (46–90)	213	NA
Michael [24]	2021	America	[^18^F]fluciclovine	Retrospective/SC	Mixed	69.79 ± 7.88	103	PET/CT (Siemens)

Note. *NA*, not available; *RP*, radical prostatectomy; *SC*, single center; *MC*, multicenter

**Table 2:** Summary of included studies using choline

Reference	Year	Country	Radiotracer	Study design	Study population	Mean/median age (y)	No. of patients	Scanner
Kwee [25]	2012	America	18F-fluorocholine	NA/SC	Mixed	69 ± 8.9	50	PET/CT (Phillips)
Schillaci [26]	2012	Italy	18F-choline	NA/SC	RP	70.9 ± 7	49	Discovery ST (GE)
Marzola [27]	2013	Italy	18F-choline	Retrospectively/SC	Mixed	69 ± 6.5	233	Discovery STE (GE)
Rybalov [28]	2013	Netherlands	11C-choline	Retrospective/SC	Mixed	69	185	PET/CT (Siemens)
Mitchell [29]	2013	America	11C-choline	Retrospective/SC	Mixed	68.8 ±7.3	176	Discovery RX or 690
Rodado-Marina [30]	2014	Spain	18F-fluorocholine	Retrospective/MC	Mixed	68 ± 7.1	233	PET/CT (Siemens)
Van-Leeuwen [31]	2015	Australia	18F-fluoromethylcholine	Prospective/SC	Mixed	68 (54–81)	38	PET/CT (Phillips)
Cimitan [32]	2015	Italy	18F-choline	Retrospective/MC	RP	69.68 ± 7.67	1000	Discovery LS (GE)
Nanni [18]	2015	Italy	11C-choline	Prospective/SC	RP	69 (55–83)	89	Discovery STE (GE)
Bluemel [16]	2016	Germany	18F-choline	Retrospective/SC	Mixed	69.4 ± 6.8	125	Biograph mCT (Siemens)
Cantiello [17]	2018	Italy	18F-choline	Retrospective/SC	RP	72 (62–82)	43	PET/CT (GE)
Michaud [33]	2020	America	11C-choline	Retrospective/SC	Mixed	67 (42–89)	287	Discovery (GE)

Note. *NA*, not available; *RP*, radical prostatectomy; *SC*, single center; *MC*, multicenter

**Table 3:** Summary of included studies using PSMA

Reference	Year	Country	Radiotracer	Study design	Study population	Mean/median age (y)	No. of patients	Scanner
Eiber [34]	2015	Germany	[^68^Ga]Ga-PSMA-11	Retrospective/SC	Mixed	70 (46–85)	248	Biograph mCT (Siemens)
Verburg [35]	2015	Germany	[^68^Ga]Ga-PSMA-11	Retrospective/SC	Mixed	70 (43–86)	155	Gemini TF16 (Philips)
van Leeuwen [36]	2016	Australia	[^68^Ga]Ga-PSMA-11	Prospective/SC	RP	62 (57–67)	70	Ingenuity TOF (Philips)
Sachpekidis [37]	2016	Germany	[^68^Ga]Ga-PSMA-11	NA/SC	Mixed	71 (54–77)	31	Biograph mCT 128 (Siemens)
Bluemel [16]	2016	Germany	[^68^Ga]Ga-PSMA-I&T	Prospective/SC	Mixed	69.4 ± 6.8	32	Biograph mCT (Siemens)
Berliner [38]	2016	Germany	[^68^Ga]Ga-PSMA-I&T	Retrospective/SC	RP	68 ± 7	83	Gemini GXL10 (Philips)
Meredith [39]	2016	Australia	[^68^Ga]Ga-PSMA-11	Retrospective/SC	Mixed	67 (44–85)	425	128 Ingenuity TF (Philips)
Mena [40]	2017	America	[^18^F]DCFBC	Prospective/SC	Mixed	68 (57–71)	68	PET/CT (Philips)
Kranzbühler [41]	2017	Switzerland	[^68^Ga]Ga-PSMA-11	Retrospective/SC	RP	69 ± 11	56	PET/MR (GE)
Schmuck [42]	2017	Germany	[^68^Ga]Ga-PSMA-I&T	Retrospective/SC	Mixed	69.8 ± 7.5	240	Biograph mCT 128 Flow (Siemens)
Afshar-Oromieh [43]	2017	Germany	[^68^Ga]Ga-PSMA-11	Retrospective/SC	Mixed	68 ± 7.8	1007	Biograph-6/Biograph mCT (Siemens)
Hope [44]	2017	America	[^68^Ga]Ga-PSMA-11	Prospective/SC	Mixed	69.0 ± 6.9	125	Discovery VCT; Signa 3.0 T PET/MRI TOF (GE, Chicago, Ill)
Dietlein [45]	2017	Germany	[^18^F]DCFPyL, [^68^Ga]Ga-PSMA-11	Retrospective/SC	Mixed	67± 6	191	Biograph mCT (Siemens)
Gupta [46]	2017	Australia	[^68^Ga]Ga-PSMA-11	Retrospective/SC	Mixed	70 (49–88)	178	Discovery-690 3D PET/CT (GE)
Sanli [47]	2017	Turkey	[^68^Ga]Ga-PSMA-11	Retrospective/SC	Mixed	71 (48–89)	109	Biograph TruePoint PET/CT (Siemens)
Kabasakal [11]	2017	Turkey	[^68^Ga]Ga-PSMA-11	Retrospective/SC	Mixed	67.34 ± 8.7	50	Biograph 6 (Siemens)
Emmett [48]	2017	Australia	[^68^Ga]Ga-PSMA-11	Prospective/SC	RP	65 (57–67)	164	Ingenuity TOF (Philips)
Habl [49]	2017	Germany	[^68^Ga]Ga-PSMA-11	Prospective/SC	RP	64 (46–79)	100	Biograph mCT (Siemens)
Medina-Ornelas [50]	2017	Mexico	[^68^Ga]Ga-PSMA-11	Retrospective/SC	Mixed	72 ± 6	84	Biograph mCT (Siemens)
Zacho [51]	2018	Denmark	[^68^Ga]Ga-PSMA-11	Prospectively/MC	Mixed	67.5 ± 6.9	70	VCT discovery True 64 (GE)
Caroli [52]	2018	Italy	[^68^Ga]Ga-PSMA-11	Prospective/SC	Mixed	69.4 ± 7.36	314	Biograph mCT Flow (Siemens)
Calais [53]	2018	America	[^68^Ga]Ga-PSMA-11	Retrospective/MC	RP	68 (43–90)	270	Various (site dependent)
Lengana [54]	2018	South Africa	[^68^Ga]Ga-PSMA-11	Prospective/SC	Mixed	66.7 ± 8.9	61	Not reported
Müller [55]	2018	Switzerland	[^68^Ga]Ga-PSMA-11	Retrospective/SC	Mixed	68 ± 6.8	223	Discovery VCT 690 (GE)
Cantiello [17]	2018	Italy	[^64^Cu]Cu-PSMA-617	Retrospective/SC	RP	72 (62–82)	43	PET/CT (GE)
De Bari [56]	2018	France	[^68^Ga]Ga-PSMA-11	Retrospective/SC	RP	69.5 (51–83)	40	Biograph mCT (Siemens)
Giesel [57]	2018	Germany	[^18^F]PSMA-1007	Retrospective/MC	Mixed	70 (48–86)	256	Biograph mCT (Siemens)
Kambiz [58]	2018	Germany	[^18^F]PSMA-1007	Retrospective/SC	Mixed	68.75 ± 7.6	100	mCT (Siemens)
Rauscher [59]	2018	Germany	[^68^Ga]Ga-PSMA-11	Retrospective/SC	Mixed	NA	272	NA
Mattiolli [60]	2018	Brazil	^68^Ga-PSMA-ligand	Retrospective/MC	Mixed	68.7 ± 8.9	125	Biograph (Siemens)
Prado Júnior [61]	2018	Brazil	^68^Ga-PSMA-ligand	NA/SC	NA	61.5 (42–94)	54	Discovery 710 (GE)
Derlin [62]	2018	Germany	[^68^Ga]Ga-THP-PSMA	Retrospective/SC	RP	70.2 ± 7.1	99	Biograph mCT 128 Flow (Siemens)
Ringheim [63]	2018	Brazil	[^68^Ga]Ga-PSMA-11	Prospective/SC	RP	67.8 ± 6.9	30	Biograph mMR (Siemens)
Gutiérrez-Cardo [64]	2018	Spain	[^68^Ga]Ga-PSMA-11	Retrospective/SC	Mixed	66 ± 7	53	Discovery STE4 (GE)
Eiber [65]	2019	Germany	[^18^F]rhPSMA-7	Retrospective/SC	Mixed	72 (49–88)	261	Biograph mCT (Siemens)
Neslihan [66]	2019	Turkey	^68^Ga-PSMA-ligand	Prospective/SC	Mixed	69 ± 8	121	Discovery ST (GE)
Hamed [67]	2019	Egypt	[^68^Ga]Ga-PSMA-11	Prospective/SC	Mixed	67.4 ± 6.9	188	Ingenuity TF 128 (Philips)
Farolfi [68]	2019	Italy	[^68^Ga]Ga-PSMA-11	Retrospective/SC	RP	66 ± 6.39	119	Discovery STE/Discovery 710 (GE)
Ceci [69]	2019	Italy	[^68^Ga]Ga-PSMA-11	Prospective/SC	Mixed	69 ± 7.1	332	Discovery 690 (GE)
Wondergem [70]	2019	Netherlands	[^18^F]DCFPyL	Retrospective/MC	Mixed	71 (67–75)	248	Ingenuity TF/ Biograph TruePoint-16 (Philips)
Asokendaran [71]	2019	Australia	[^68^Ga]Ga-PSMA-I&T	Retrospective/SC	Mixed	68.5 (45–84)	150	Discovery 710 (GE)
Bashir [72]	2019	Britain	[^68^Ga]Ga-PSMA-11	Retrospective/SC	Mixed	65.6 (50–76.2)	28	Gemini (Philips)
Beheshti [73]	2019	Germany	[^68^Ga]Ga-PSMA-11	Prospective/SC	Mixed	66.8 ± 8.0	135	Discovery 710 (GE)
Song [74]	2019	America	[^18^F]DCFPyL	Prospective/SC	Mixed	71.5 ± 7.2	72	Discovery MI (GE)
Hoffmann [10]	2019	Germany	[^68^Ga]Ga-PSMA-11	Retrospective/MC	Mixed	70 ± 8	660	Gemini TF16 (Phillips)
Kulkarni [75]	2020	Britain	[^68^Ga]Ga-THP-PSMA	NA	Mixed	68.2 (49–85)	68	Discovery 710 (GE)
Seniaray [76]	2020	India	[^68^Ga]Ga-PSMA-11	Retrospective and prospective/SC	Mixed	68 ± 6.4	170	NA
Perry [77]	2021	New Zealand	[^18^F]DCFPyL	Retrospective/MC	RP	71 (49–89)	222	Discovery 710 (GE) Discovery 690 (GE)

### Publication bias and heterogeneity and quality assessment

A symmetrical funnel-shaped distribution of PSA < 0.5 ng/mL (*p* = 0.96) and PSA levels of 0.5–1.0 ng/mL (*p* = 0.12) indicated that there was no significant publication bias. However, publication bias was found in the cohorts with PSA levels of 1–1.99 ng/mL (*p* < 0.001). The Egger test was used to quantify significant asymmetry.

**Fig. 3:**
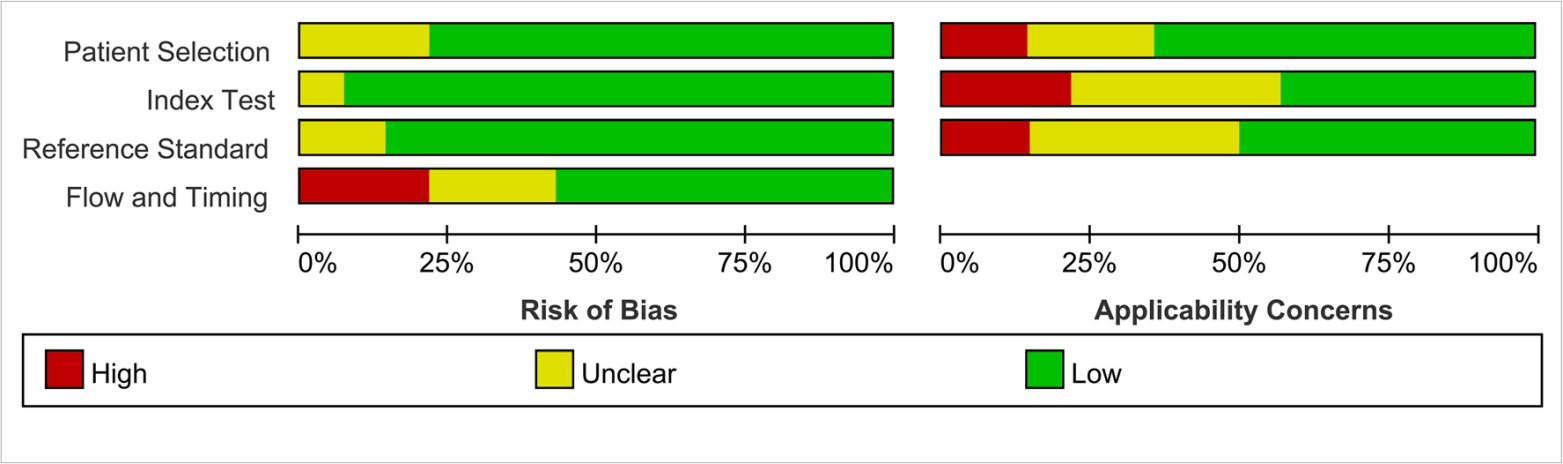
Depiction of the study quality assessment using the QUADAS2 tool

The forest plots revealed strong heterogeneity for the fluciclovine cohort, as the *I*^2^ was 64%, 70%, and 72% for a PSA level < 0.5 ng/mL (*p* < 0.05), 0.5–0.9 ng/mL (*p <* 0.01), and 1.0–1.99 ng/mL (*p <* 0.01), respectively. The *I*^2^ was 78%, 66%, and 78% for a PSA level < 0.5 ng/mL (*p <* 0.01), 0.5–0.9 ng/mL (*p <* 0.01), and 1.0–1.99 ng/mL (*p <* 0.01), respectively, in the choline cohort. *I*^2^ values of the overall pooled DR were 81%, 78%, and 75% for a PSA level < 0.5 ng/mL (*p <* 0.01), 0.5–0.9 ng/mL (*p <* 0.01), and 1.0–1.99 ng/mL (*p <* 0.01), respectively, in the PSMA cohort. The quality assessment of the included studies is shown in Fig. 3. QUADAS-2 revealed that the majority of included studies were at a moderate risk of bias. Because all studies had consistent qualified patient selection criteria, patient selection was not considered the major potential source of bias. For the reference standard, some articles were marked as unclear or high levels because of the lack of consistent reference standards and clinical follow-up times.

### Detection rates of choline, [^18^F]fluciclovine, and PSMA PET/CT

**Fig. 4:**
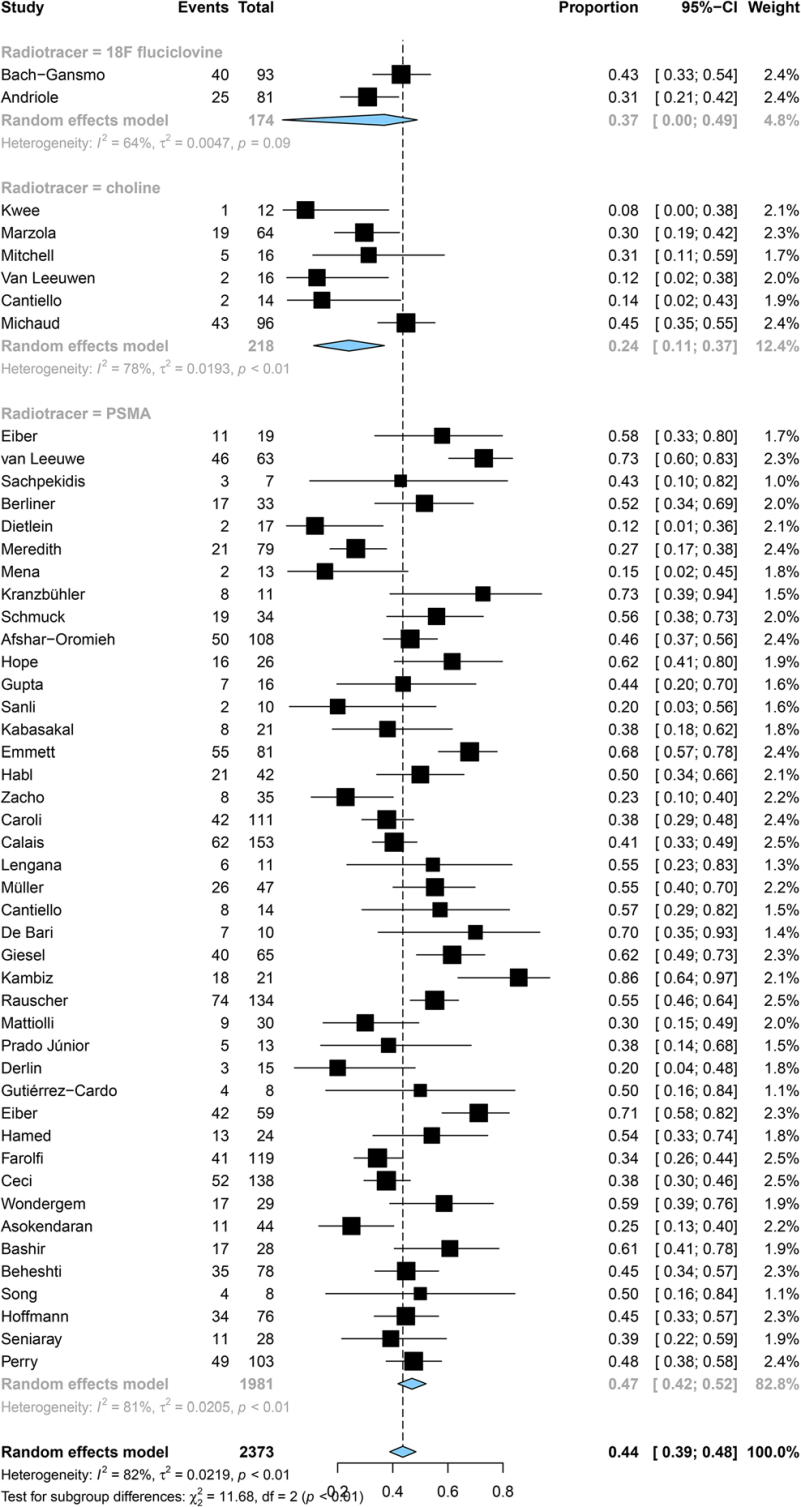
Forest plot of the proportion of choline, fluciclovine, and PSMA positivity of prostate cancer patients with BCR for PSA less than 0.5 ng/mL

**Fig. 5:**
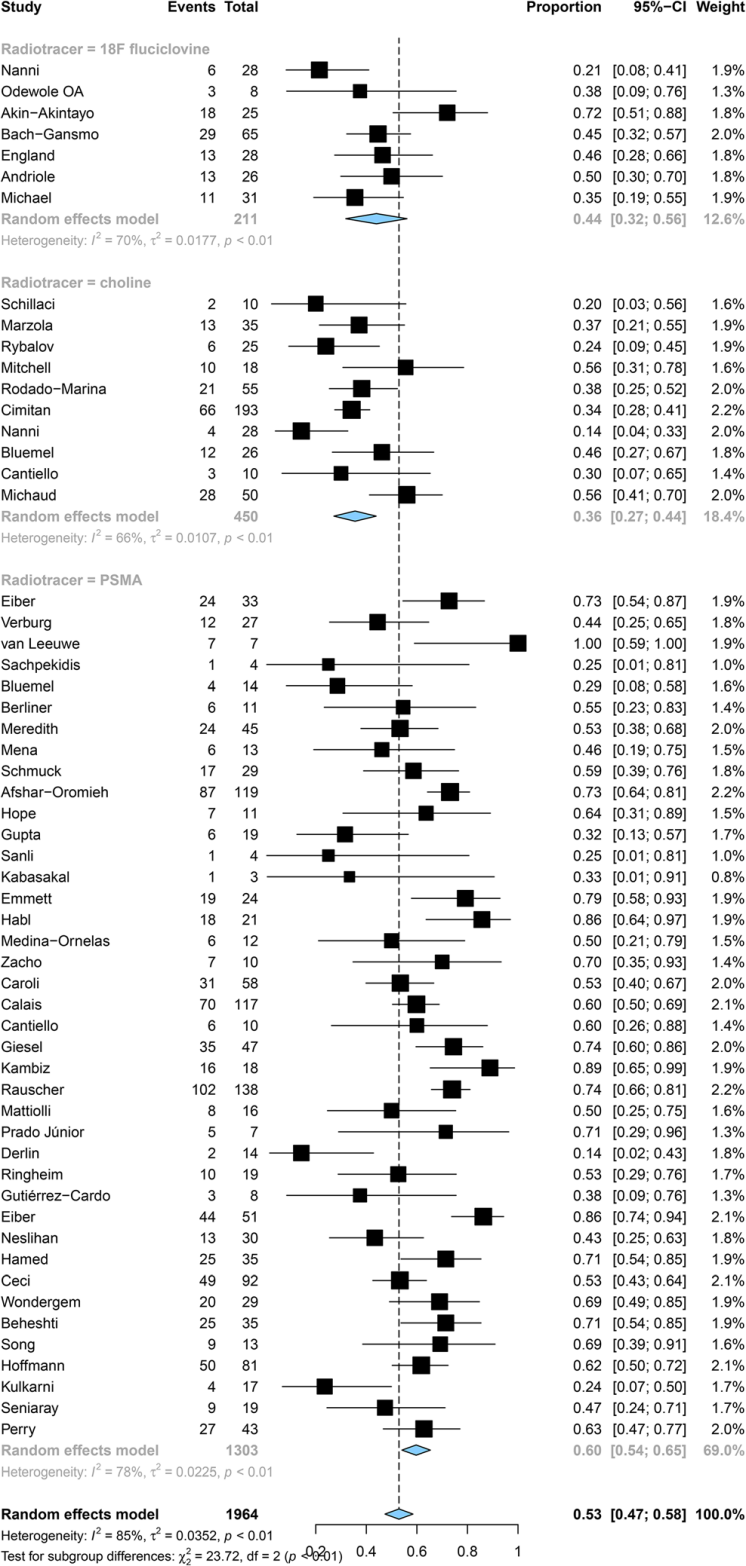
Forest plot of the proportion of choline, fluciclovine, and PSMA positivity of prostate cancer patients with BCR for PSA 0.5–0.99 ng/mL

**Fig. 6:**
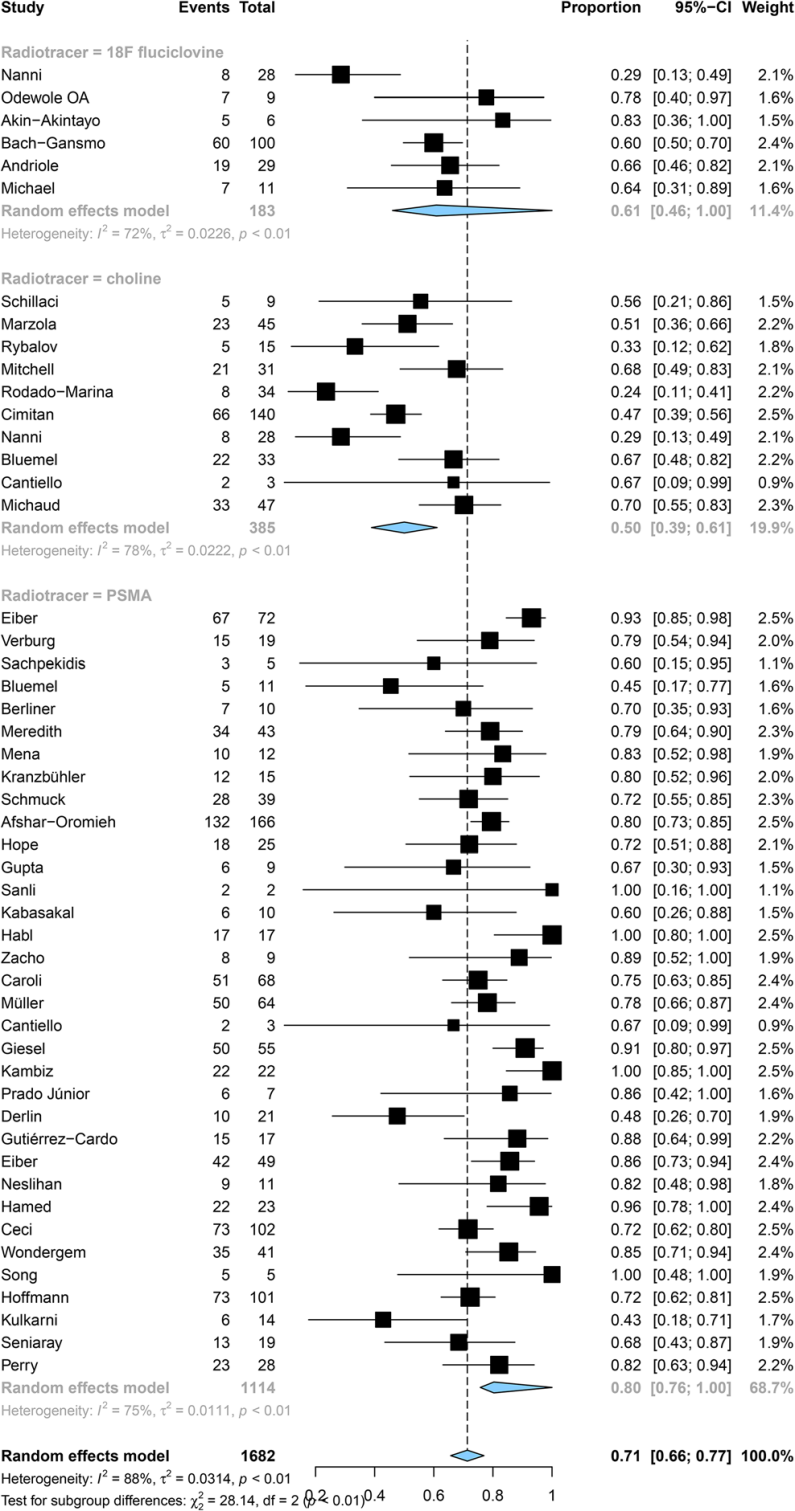
Forest plot of the proportio.n of choline, fluciclovine, and PSMA positivity of prostate cancer patients with BCR for PSA 1.0–1.99 ng/mL

Pooled DRs of choline, ^18^F-fluciclovine, and PSMA were 24% (95% CI: 11%, 37%), 37% (95% CI: 0%, 49%), and 47% (95% CI: 42%, 52%) for PSA levels < 0.5 ng/mL (*p <* 0.001), respectively (Fig. 4); 36% (95% CI: 27%, 44%), 44% (95% CI: 32%, 56%), and 60% (95% CI: 54%, 65%) for a level of 0.5–0.9 ng/mL (*p <* 0.001) (Fig. 5); and 50% (95% CI: 39%, 61%), 61% (95% CI: 46%, 100%), and 80% (95% CI: 76%, 100%) for a level of 1–1.99 ng/mL (*p* < 0.001), respectively (Fig. 6).

### Comparison of ^18^F-labeled vs ^68^Ga-labeled PSMA studies

Table 4 shows the results of the point estimates of the pooled DRs for the difference between ^18^F-labeled and ^68^Ga-labeled PSMA. The DR with ^18^F-labeled PSMA was higher than that with ^68^Ga-labeled PSMA. In addition, the DR showed an increasing magnitude with an increase in the PSA level, which was significant for all PSA levels.

**Table 4:** Point estimates of the mean difference between 68Ga-labeled PSMA and 18F-labeled PSMA detection rates (DRs) according to PSA level

PSA level	18F-DR (%)	68Ga-DR (%)	Difference in DRs (%)	*p*-value
< 0.5 ng/mL	58	44	14	< 0.01
0.5–0.99 ng/mL	72	56	16	< 0.01
1.0–1.99 ng/mL	88	78	10	< 0.01

## Discussion

Whereas other meta-analyses have investigated the diagnostic roles of tracers applied in PCa with BCR [2, 6, 7, 78, 79], to our knowledge, this is the first comparative meta-analysis that focuses on all three relevant tracers for the early detection of this disease. This meta-analysis showed a higher pooled DR for PCa with BCR using PSMA compared to that with fluciclovine and choline PET/CT for three PSA levels, and we observed a significant difference. These results are in accordance with a previous meta-analysis that included only ^18^F-labeled choline, fluciclovine, and PSMA, which reported that PSMA was better than choline and fluciclovine [6]. A meta-analysis recently showed that there is a trend but no significant difference when the PSA level is < 0.5 ng/mL and 0.5–0.9 ng/mL. However, our study found an absolute statistical difference when comparing the DRs of PSMA and fluciclovine. A possible reason for this is the limited number of studies. In general, PET/CT imaging is more likely to be negative with low PSA values.

Radiation therapy remains the gold standard for intermediate- and high-risk, localized prostate cancer. While these are effective forms of management, approximately 30–40% of cancers still recur following treatment, manifesting as a rising prostate-specific antigen (PSA). The key issue for patients with BCR is the early and correct identification of recurrent or metastatic disease. Conventional imaging modalities consisting of CT, bone scan, and MRI have been used for patients with PCa, but their diagnostic performance in detecting minimal or occult lesions is limited. At this stage of the disease, it is important to determine the location and extent of metastases to determine the next course of management. PET is an established, non-invasive, molecular imaging modality that uses different radiolabeled tracers, a combination of a radionuclide and a biologically active molecule, targeted to specific receptors to localize disease. PET/CT has a higher detection rate of intra-prostatic tumors that might have clinical implications regarding focal therapy such as radiotherapy and surgical planning. The current study has demonstrated that PSMA-based tracer PET/CT imaging seems to be a promising tool and shows clear superiority in the detection of PCa with BCR and PSA when compared to choline- and fluciclovine-based tracers. In clinical practice, choline PET/CT is the most commonly used radioactive tracer [17]. Significantly, choline PET/CT exhibits a higher DR only at high PSA levels [80]. A previous study has shown that the DR of PCa with BCR and PSA < 1.5 ng/mL was only below 30% when using choline-based tracers PET/CT [81, 82], which is in accordance with our findings.

In our study, ^18^F-fluciclovine also showed a higher DR than choline (37% vs 24% for 0.2–0.5 ng/mL, 44% vs 36% for 0.5–1.0 ng/mL). Similarly, in a prospective study, the authors showed that the overall performance with [^18^F]FACBC, a relatively new radiotracer, was higher than that of ^11^C-choline, and they found that this difference in DR was particularly significant for PCa with BCR and a PSA level < 1 ng/mL^18^. Furthermore, current EAU guidelines recommend that choline PET/CT only be used for non-early PCa recurrence with serum PSA levels > 1 ng/mL [83]. Afshar-Oromieh et al calculated the detection rate of [^68^Ga]Ga-PSMA-11 to be 46% (32/69) for PSA < 0.2 ng/mL, 46% (50/108) for 0.2–0.5 ng/mL, and 73% (87/119) for 0.5–1.0 ng/mL [43]. In addition, a higher detection rate of PCa with BCR with low PSA levels was suggested by other research [59]. Considering these aspects, PSMA should be the preferred tracer choice, especially for patients with low PSA (≤ 2.0 ng/mL).

However, PSMA PET/CT is an increasingly used tracer for patients with BCR and achieves a high DR for early PCa recurrence (PSA ≤ 2.0 ng/mL) [84]. Furthermore, the strength of evidence was limited by publication bias, multiple reference standards, and a lack of consistent clinical follow-up times. In particular, our meta-analyses also performed subgroup analyses based on ^18^F-labeled and ^68^Ga-labeled PSMA. We observed statistically significant differences when comparing ^18^F-labeled and ^68^Ga-labeled PSMA (PSA < 0.5 ng/mL: 58% vs 44%, *p* < 0.01; PSA level 0.5–1.0 ng/mL: 72% vs 56%, *p* < 0.01; PSA 1.0–1.99 ng/mL: 88% vs 78%, *p* < 0.01). To date, to our knowledge, this is the first evidence-based study to evaluate the DRs of ^18^F-labeled and ^68^Ga-labeled PSMA. Recently, there have been multiple meta-analyses showing that the summary DR of ^18^F-labeled PSMA in patients with BCR was approximately 49% for PSA < 0.5 ng/mL [1, 78, 85], which is slightly better than the 44.9% detection rate of ^68^Ga-PSMA in a recent prospective study [73]. Compared to ^18^F-PSMA, ^68^Ga-PSMA ligands have a short half-life (68 min), and thus are inconvenient for transport [86]. Moreover, they are characterized by a lower signal-to-noise ratio for images [87], limiting its clinical application in detecting occult or metastatic lesions in the prostate bed. However, ^18^F-PSMA analogs seemed to be more favorable due to their longer half-life and a higher physical spatial resolution, and [^18^F]PSMA-1007, as a second-generation ^18^F-labeled PSMA tracer, demonstrated high labeling yields, better tumor uptake, and hepatobiliary excretion, making it an ideal PSMA-target tracer for diagnostic imaging in patients with BCR. Accordingly, this might explain why some authors considered [^18^F]DCFPyL to be a good replacement for recurrent PCa.

Our meta-analysis has several limitations. First, significant heterogeneity was observed in all cohorts. Second, because the sample size was limited, retrospective, single-institutional studies accounted for a large amount, which might be one of the reasons for the selection bias. Additionally, the different PET/CT scanners, radiotracers, patient populations, and various treatment modes increased the risk of bias and significant heterogeneity.

In conclusion, our meta-analysis revealed that PSMA-radiotracers demonstrate a potentially promising DR with low PSA levels in biochemically recurrent PCa. PSMA has a relatively higher DR than fluciclovine and choline in PCa patients with BCR and with PSA < 2.0 ng/mL. Additionally, 18F-labeled PSMA achieved a higher DR than ^68^Ga-labeled PSMA.

## Abbreviations

BCR: Biochemical recurrence
CT: Computed tomography
DR: Detection rate
MRI: Magnetic resonance imaging
PCa: Prostate cancer
PSA: Prostate-specific antigen
PSMA: Prostate-specific membrane antigen

## Declarations

### Guarantor

The scientific guarantor of this publication is Jiwei Mao.

### Statistics and biometry

One of the authors performed statistical analysis for this paper.

### Methodology

• systematic review

• meta-analysis
